# Tumor-associated macrophages respond to chemotherapy by detrimental transcriptional reprogramming and suppressing stabilin-1 mediated clearance of EGF

**DOI:** 10.3389/fimmu.2023.1000497

**Published:** 2023-03-07

**Authors:** Irina Larionova, Artem Kiselev, Elena Kazakova, Tengfei Liu, Marina Patysheva, Pavel Iamshchikov, Quan Liu, Dieuwertje M. Mossel, Vladimir Riabov, Militsa Rakina, Alexey Sergushichev, Natalia Bezgodova, Sergei Vtorushin, Nikolai Litviakov, Evgeny Denisov, Philipp Koshkin, Denis Pyankov, Matvei Tsyganov, Marina Ibragimova, Nadezhda Cherdyntseva, Julia Kzhyshkowska

**Affiliations:** ^1^ Laboratory of translational cellular and molecular biomedicine, National Research Tomsk State University, Tomsk, Russia; ^2^ Cancer Research Institute, Tomsk National Research Medical Center, Russian Academy of Sciences, Tomsk, Russia; ^3^ Laboratory of Genetic Technologies, Siberian State Medical University, Tomsk, Russia; ^4^ Institute for Quantitative Health Science and Engineering (IQ), Michigan State University, East Lansing, MI, United States; ^5^ Institute of Transfusion Medicine and Immunology, Mannheim Institute for Innate Immunoscience (MI3), Medical Faculty Mannheim, University of Heidelberg, Mannheim, Germany; ^6^ Saint Petersburg National Research University of Information Technologies, Mechanics and Optics (ITMO University), Saint Petersburg, Russia; ^7^ Laboratory of Molecular Pathology, Genomed, Moscow, Russia; ^8^ German Red Cross Blood Service Baden-Württemberg – Hessen, Mannheim, Germany

**Keywords:** tumor-associated macrophage (TAM), cisplatin, breast cancer, stabilin-1, EGF, endocytosis, clearance

## Abstract

**Introduction:**

Tumor resistance to chemotherapy and metastatic relapse account for more than 90% of cancer specific mortality. Tumor-associated macrophages (TAMs) can process chemotherapeutic agents and impair their action. Little is known about the direct effects of chemotherapy on TAMs.

**Methods:**

The effect of chemotherapeutic platinum agent cisplatin was assessed in the model system of human ex vivo TAMs. Whole-transcriptome sequencing for paired TAMs stimulated and not stimulated by cisplatin was analysed by NGS. Endocytic uptake of EGF was quantified by flow cytometry. Confocal microscopy was used to visualize stabilin-1-mediated internalization and endocytic trafficking of EGF in CHO cells expressing ectopically recombinant stabilin-1 and in stabilin-1+ TAMs. In cohort of patients with breast cancer, the effect of platinum therapy on the transcriptome of TAMs was validated, and differential expression of regulators of endocytosis was identified.

**Results:**

Here we show that chemotherapeutic agent cisplatin can initiate detrimental transcriptional and functional programs in TAMs, without significant impairment of their viability. We focused on the clearance function of TAMs that controls composition of tumor microenvironment. For the first time we demonstrated that TAMs’ scavenger receptor stabilin-1 is responsible for the clearance of epidermal growth factor (EGF), a potent stimulator of tumor growth. Cisplatin suppressed both overall and EGF-specific endocytosis in TAMs by bidirectional mode: suppression of positive regulators and stimulation of negative regulators of endocytosis, with strongest effect on synaptotagmin-11 (SYT11), confirmed in patients with breast cancer.

**Conclusion:**

Our data demonstrate that synergistic action of cytostatic agents and innovative immunomodulators is required to overcome cancer therapy resistance.

## Introduction

Therapeutic sensitivity of tumors substantially depends on the complex interaction of transformed cells with various components of the tumor microenvironment (TME) (1). Tumor is a complex system of transformed cells interacting with the surrounding heterogeneous cellular and molecular microenvironment, which affects the key properties of cancer cells and contribute to the malignance progression and its response to therapy (2). In turn, malignant cells alter the phenotype and function of infiltrating cells and stroma to survive and escape from immune surveillance (3, 4).

Tumor-associated macrophages (TAMs) are the major component of innate immunity of TME that enhance tumor growth and angiogenesis, stimulate the invasion of tumor cells and metastasis, control immunosuppression and response to therapy (5, 6). In major tumor types, TAMs have pro-tumor M2 orientation, and correlate to poor prognosis and metastasis (7). Data generated in the international cohorts of breast cancer patients demonstrated the positive correlation of TAMs with parameters of tumor progression including lymphatic and hematogenous metastasis, recurrence, and survival (7, 8).

In breast cancer, TAMs are represented by phenotypes, expressing the variety of scavenger receptors (CD163, CD204, CD206 and stabilin-1) (7). Scavenger receptors (SRs) are essential biomarkers of TAMs (9). Their ligands include modified LDL, phospholipids, apoptotic cells, amyloid proteins, ferritin, hyaluronan (HA), heparin, and matricellular protein (SPARC) (9–11). TAMs internalize different endogenous factors *via* endocytosis resulting in altering extracellular landscape of TME and regulating tumor development (12, 13).

Macrophages induce chemotherapy (CT) resistance and tumor progression after treatment by activating tumor revascularization, suppressing cytotoxic T cell immunity and activating the anti-apoptotic program in tumor cells (14). The accumulation of TAMs in breast tumors after CT was identified in both mouse models and cancer patients (14). While most mouse models demonstrated the negative role of TAMs in the tumor response to CT, there is still no consensus about the effect of TAMs on the efficiency of chemotherapy in patients with breast cancer (7). The mechanisms of the direct action of chemotherapeutic drugs on TAMs in breast cancer is an open question.

Understanding the mechanisms of interaction between TAMs and CT agents is urgently needed to predict the effectiveness of chemotherapy and to develop therapeutic regimens that enhance the antitumor activity of TAMs. Markers involved in the tumor response to chemotherapy can be used as targets for immunomodulation to increase the effectiveness of the treatment (15).

In our study, we analyzed the response of TAMs to cisplatin, an antitumor drug that belongs to platinum-based therapy applied in breast cancer patients (16). The mechanism of its action is related to the formation of DNA crosslinks both between and within DNA strands (known as interstrand and intrastrand cross-linkages, respectively), that is irreversible and leads to cell apoptosis (16). We made systemic analysis of cisplatin effect on transcriptional profile of TAMs, identified that cisplatin-treated TAMs activate several programs that can support tumor progression, and in details investigated the effect of cisplatin on the mechanism of suppression of receptor-mediated scavenging function of TAMs.

## Materials and methods

### Patients

The study included female patients with invasive breast carcinoma of no special type with morphologically verified diagnosis, treated in clinics of Cancer Research Institute of Tomsk National Research Medical Center (Tomsk, Russia). The study was carried out according to Declaration of Helsinki (from 1964, revised in 1975 and 1983) and was approved by the local committee of Medical Ethics of Tomsk Cancer Research Institute; all patients signed informed consent for the study. For IHC analysis, patients with Ia-IIIb clinical stages (T1-4N0-1M0) were divided into two groups according to the neoadjuvant treatment: 1) patients who did not receive neoadjuvant chemotherapy (NACT) (N=16) and 2) patients underwent cisplatin-based NACT (N=11). Patients with NACT received 6-8 courses of chemotherapy in accordance with the «Primary breast cancer: ESMO Clinical Practice Guidelines for diagnosis, treatment and follow-up 2015” (17). Chemotherapeutic regimens included CP (cisplatin plus cyclophosphamide) and CAP (cyclophosphamide, adriamycin and platinum). Patients` characteristics are presented in **Supplementary Table S1**.

The microarray study included six patients with T1-2N0-1M0 breast cancer (stage IIA – IIB). The material for the study was paired samples of biopsy material before treatment and surgical material after NACT for each of the patients. All patients had a BRCA1 gene deletion in their tumors and therefore received systemic NACT based on CP. The luminal B subtype of breast cancer was defined as ER+, PR+ or -, and Ki67 > 30%, and all patients with the luminal B subtype had HER2-negative status.

In adjuvant regime, patients underwent radiation therapy and/or hormone therapy after the surgery. Hormone therapy was prescribed to all patients with the luminal B subtype. Radiation therapy was prescribed in the presence of lymphatic metastases.

### Cell lines

MCF-7 (breast adenocarcinoma) cells purchased from ATCC (KCB Cat# KCB 200831YJ, RRID : CVCL_0031) were cultured in complete DMEM media (Gibco, USA), containing 10% FCS and 1% penicillin/streptavidin. During the cultivation of tumor cells, the medium of each cell line was changed to 2% serum containing medium for 36 hours to minimize the effect of serum on monocyte differentiation. Supernatants from serum-starved tumor cells were harvested and filtered through low protein-binding filters (ROTH, Germany). Fresh supernatants and medium were added to freshly isolated monocytes in amount of 20%.

Chinese hamster ovary (CHO) cells (KCB Cat# KCB 83004YJ, RRID : CVCL_0213) stably transfected with empty vector (CHO-V3a) and full-length human stabilin-1 expressed constructs (CHO-Stab1) were generated as described (18). CHO-V3a and CHO-Stab1 (clone P2F11) cells were propagated in Ham’s F12 medium supplemented with 10% FBS and 1% penicillin/streptavidin.

### Monocytes isolation and generation of *ex vivo* TAMs

Human monocytes were obtained from the German Red Cross Blood Service Baden-Württemberg–Hessen (Mannheim, Germany) and from Tomsk Regional Blood Center (Tomsk, Russia). German cohort of donors was used for the real-time PCR and NGS, Russian cohort – for the flow cytometry, viability assay and confocal microscopy. Monocytes were isolated from the buffy coats of healthy donors by density gradients followed by positive magnetic selection using CD14+ MACS beads (no. 130-050-201, Miltenyi Biotech, Germany), resulting to 90–98% monocyte purity as confirmed by flow cytometry. Monocytes were cultured at the concentration of 10^6 cells/ml in serum-free X-VIVO medium (Lonza, Germany) supplemented with 10 ng/ml of macrophage colony-stimulating factor (M-CSF) (no. 300-25, Peprotech, Germany), 10−8 M of dexamethasone (no. D2915, Sigma-Aldrich, Germany) and 10 ng/ml of IL-4 (no. 200-04, Peprotech, Germany). Supernatants from MCF-7 cells were added to freshly isolated human primary monocytes at a final volume of 20% of a cultivation medium. Monocytes were differentiated to TAMs for 6 days in the presence of 7.5% CO2.

### Cisplatin treatment

Cisplatin (Cisplatin-Teva, Teva Pharmaceutical Industries, Ltd. L01XA01 injection solution) was used for chemotherapeutic treatment of TAMs *in vitro*. Cisplatin experimental concentrations were first chosen based on the half-maximal inhibitory concentration dose 50 (IC50) for cancer cell line MCF-7. For *ex vivo* TAMs, cisplatin was used at the selected concentrations (20 µM, 40 µM, and 80 µM) to determine the optimal concentration for further analysis. Viability test and apoptosis assay demonstrated that cisplatin decreased the survival of macrophages in dose-dependent manner: at concentration of 80 µМ the viability was 10%, at 40 µМ - 30%, at 20 µМ – 50-80% (data not shown). The 20 µМ concentration of cisplatin was used for further analysis. It was added on day 6 of macrophages differentiation and TAMs were incubated with cisplatin for 3 days.

### Viability assay

The viability of *ex vivo* TAMs was analyzed by alamarBlue^®^ Cell Viability Assay (no. DAL1025, ThermoFisher). We added 10x alamarBlue cell viability reagent as 10% of the sample volume, followed by 3 hours’ incubation at 37°C. The resulting fluorescence was read on Cytation™ 1 Cell Imaging Multi-Mode Reader (BioTek). Color change was detected using absorbance (detected at 570 and 600 nm).

### Endocytosis assay

Endocytosis was performed in *ex vivo* TAMs and in CHO cells. Ligands, AlexaFluor488-AcLDL (L23380, Low density lipoprotein, acetylated, AlexaFluor488 conjugate, Life Technologies, USA) and AlexaFluor488-EGF (no. E13345, Epidermal Growth Factor, biotinylated, complexed to AlexaFluor 488 Streptavidin, Life Technologies, USA), were added for 30 min (the concentration for each ligand is 2 µg/mL). To stop endocytosis cells were placed on ice and then quickly washed three times with PBS followed by a quantitative analysis of the ligand internalization by flow cytometry.

### Flow cytometry

Quantification of bound/internalized fluorescent ligands was performed with FACSCanto II flow cytometer (BD Biosciences) for CHO cells and CytoFLEX (Beckman Coulter) for TAMs using standard protocols. The control sample without labeled ligand was used for each stimulation. To assess EGF and acLDL endocytosis, the gate was set on the whole population of macrophages excluding cellular debris, based on FSC/SSC scatter pattern. Data were visualized and analyzed using FlowJo 7.6.5 software (FlowJo, RRID : SCR_008520) and CytExpert Software 2.0 (CytExpert Software, RRID : SCR_017217), respectively.

### Cytospin preparation

Macrophages were harvested by scraping on ice and used for cytospin preparation (2×105 cells per cytospin) using Thermo Scientific Shandon Cytospin^®^ 4 Cytocentrifuge. Cytospins were fixed for 10 min in 2% PFA in PBS, permeabilized for 15 min in 0.5% Triton X-100 in PBS, and fixed for 10 min with 4% PFA in PBS, then washed 3 times in PBS. Cytospins were stored at -80 °С. CHO cells were cultivated on coverslips to confluence between 70 and 90%. All fixation and staining procedures were performed at room temperature.

### Quantitative immunohistochemical analysis of breast cancer samples

Formalin fixed paraffin embedded (FFPE) tissue sections were obtained from breast cancer patients. The antigen unmasking was performed using the PT Link module (Dako, Denmark) in T/E buffer (pH 9.0). Immunohistochemical staining was performed using monoclonal mouse anti-CD68 (1:100, NBP2-44539, clone KP1, Novus Biologicals), polyclonal goat anti-CD206 (1:40, AF2534, RD systems) and polyclonal rabbit anti-stabilin-1 antibodies, generated by us (19), and visualized using Polymer-HRP detection system (ab236466, Abcam, USA). The staining results were acquired by Carl Zeiss Axio Lab.A1 light microscope (Jenamed, Carl Zeiss, Germany). Tissue scanning was performed with Leica Aperio AT2 (Leica, Germany) and ScanScope software (Aperio ScanScope XT Leica, RRID : SCR_018457). The histoscanning results were processed using the QuPath 0.2.3 software (RRID : SCR_018257). The intensity and the proportion of positive cells were estimated and the H-score was obtained for each slide. H-score parameter was automatically calculated using intensity thresholds that were set to further subclassify cells as being negative, weak, moderate or strongly positive.

### Immunofluorescence and confocal microscopy

For immunofluorescence (IF) staining on cytospins, samples were blocked with 3% BSA in PBS for 45 min, incubated with a combination of primary antibodies for 1,5 h; washed, and incubated with a combination of appropriate secondary antibodies for 45 min. Anti-stabilin-1 rabbit polyclonal antibody (RS1) (20) was used at 1:800 dilution; anti-EEA1 mouse monoclonal antibody (clone 14/EEA1, cat #610457, BD bioscience, Germany) was used at 1:500 dilution; mouse monoclonal antibody anti-Lamp1 (Novus Biologicals, NBP-2-52721) was used at 1:200. Following combinations of secondary antibodies were used: (a) Cy5-conjugated anti-rabbit and Cy3-conjugated anti-mouse antibodies for IF staining in *ex vivo* TAMs; (b) Cy5-conjugated anti-mouse and Cy3-conjugated anti-rabbit antibodies (all donkey, Dianova, Germany, dilution 1:400) in CHO cells. Samples were mounted with Fluoroshield Mounting Medium (ab104135 Abcam, USA) and analyzed by confocal microscopy. Analysis of EGF/stabilin-1/EEA1 co-localization in TAMs non-treated and treated with cisplatin was performed using Carl Zeiss LSM 780 NLO laser scanning spectral confocal microscope (Carl Zeiss, Germany), equipped with a 40x and 63x objectives. 3D reconstruction of the EGF/stabilin-1/EEA1 co-localization was performed using Z-stack images and Black Zen software. Images for CHO cells and for EGF/stabilin-1/Lamp1 co-localization in TAMs were acquired using Leica TCS SP8 laser scanning spectral confocal microscope (Leica Microsystems, Germany). A 63x objective lens with a numerical aperture of 1.32 for immersion with oil was used. Data were acquired and analyzed with Leica confocal software and Black Zen software (RRID : SCR_018163), respectively. All three-color images were acquired using a sequential scan mode.

### DNA and RNA isolation

RNA isolation was performed with E.Z.N.A. Total RNA Kit (Omega Bio-Tek, USA) and RNeasy Plus Mini Kit (Qiagen, Germany). The cells were lysed in Lysis Buffer with beta-mercaptoethanol, and centrifuged on columns with wash buffers. RNA was eluted with RNAase-free DEPC-water. The concentration of RNA was measured on Qubit 3.0, the quality of RNA was determined by capillary electrophoresis on TapeStation (AgilentTechnologies, USA) and using R6K ScreenTape (Agilent Technologies, USA #5067-5367). RIN was 6,7-9,8.

DNA for microarray analysis was isolated using the QIAamp DNA Mini Kit (Qiagen, Germany).

### Quantitative real-time PCR analysis

RNA was isolated out of TAMs on day 9 of cultivation. The gene expression was measured by quantitative real-time PCR using the Taqman technology and was normalized to the expression of housekeeping gene glyceraldehyde 3-phosphate dehydrogenase (GAPDH). The amplification of IL-1b, IL-6, IL-8 (CXCL8), TNFa, TGFb, VEGFA, CHI3L1 (YKL-40), CHI3L2 (YKL-39), STAB1, (MRC1) CD206, CD163, and CD36 was performed with Light Cycler 480 Real-Time PCR system (Roche, Germany) using standard conditions. Primer and probed was obtained from Life Technologies. The amplification of DNM3, STX8, DENND1A, SYT11, SCAMP5 and RUBCN was performed using AriaMix Real-Time PCR system (Agilent Technologies, USA). Primers and probes (FAM-BHQ1) were selected using Vector NTI Advance 11.5 program and the NCBI database (http://www.ncbi.nlm.nih.gov/nuccore) and were synthesized by DNA-synthesis Company (Moscow, Russia). The self-designed primer sequences are listed in **Supplementary Table S2**.

### RNA sequencing and data analysis

RNA libraries were prepared with NEXT flex Rapid Directional qRNA-SeqKit using indexed barcodes NEXTflex-qRNA-8nt-Barcodes (NOVA-5198-02, Bioo Scientific, PerkinElmer Applied Genomics, USA) according manufacture`s protocols. Ribosomal RNA depletion was performed with NEBNext^®^ rRNA Depletion Kit (Human/Mouse/Rat) ((NEB #E7400, New England Biolabs Inc., USA).

Whole-transcriptome sequencing was performed in macrophages obtained from 3 donors. Prepared libraries were then pooled and sequenced on an Illumina NextSeq500 instrument (Illumina, USA) with NextSeq 500/550 High-Output v2.5 Kit (75 cycles) (cat #20024906). Raw data quality was obtained using FastQC software (FastQC, RRID : SCR_014583) (https://www.bioinformatics.babraham.ac.uk/projects/fastqc/). Data analysis was performed using STAR aligner (STAR, RRID : SCR_004463) (21), with GRCh38 genome and Gencode annotations (22). The numbers of reads assigned to features (exons of coding genes) were calculated using featureCounts software (featureCounts, RRID : SCR_012919) (23). Subsequent analysis steps were performed using DESeq2 software (DESeq, RRID : SCR_000154), part of Bioconductor project (Bioconductor, RRID : SCR_006442) (24). Differential expression data was visualized and analyzed with Phantasus software (https://genome.ifmo.ru/phantasus). Fgsea software (fgsea, RRID : SCR_020938) (https://www.biorxiv.org/content/early/2016/06/20/060012) was used for gene set enrichment analysis of biochemical and regulatory pathways using gene lists ranked by expression level and p value. Reaсtome database was used *via* reactome.db R package.

### Microarray analysis

The presence of CNA (Copy Number Aberrations) before and after NACT was determined using a CytoScan HD Array microarray (Affymetrix, USA). Gene expression was evaluated using a Human Clariom S Assays microarray (Affymetrix, USA), Chromosome Analysis Suite 4.0 (ChAS, RRID : SCR_015626, Affymetrix, USA) and Transcriptome Analysis Console (TAC) 4.0 software (RRID : SCR_018718), respectively, were used to process the results of microchipping (bioinformatic analysis).

### Statistical analysis

Statistical analysis was performed using STATISTICA 8.0 for Windows (STATISTICA, RRID : SCR_014213). The Chi-square test and Manna-Whitney test were implemented. Results of real-time PCR and flow cytometry analysis were presented using GraphPad Prism 6 software (GraphPad Prism, RRID : SCR_002798). Results were considered to be significant with ***p<0,001, ** p<0,01 and * p<0,05. Data with marginal significance (p value <0.1) were also discussed.

## Results

### Cisplatin-based chemotherapy does not decrease the amount of macrophages in remaining tumor tissue of patients with breast cancer

**Figure 1:**
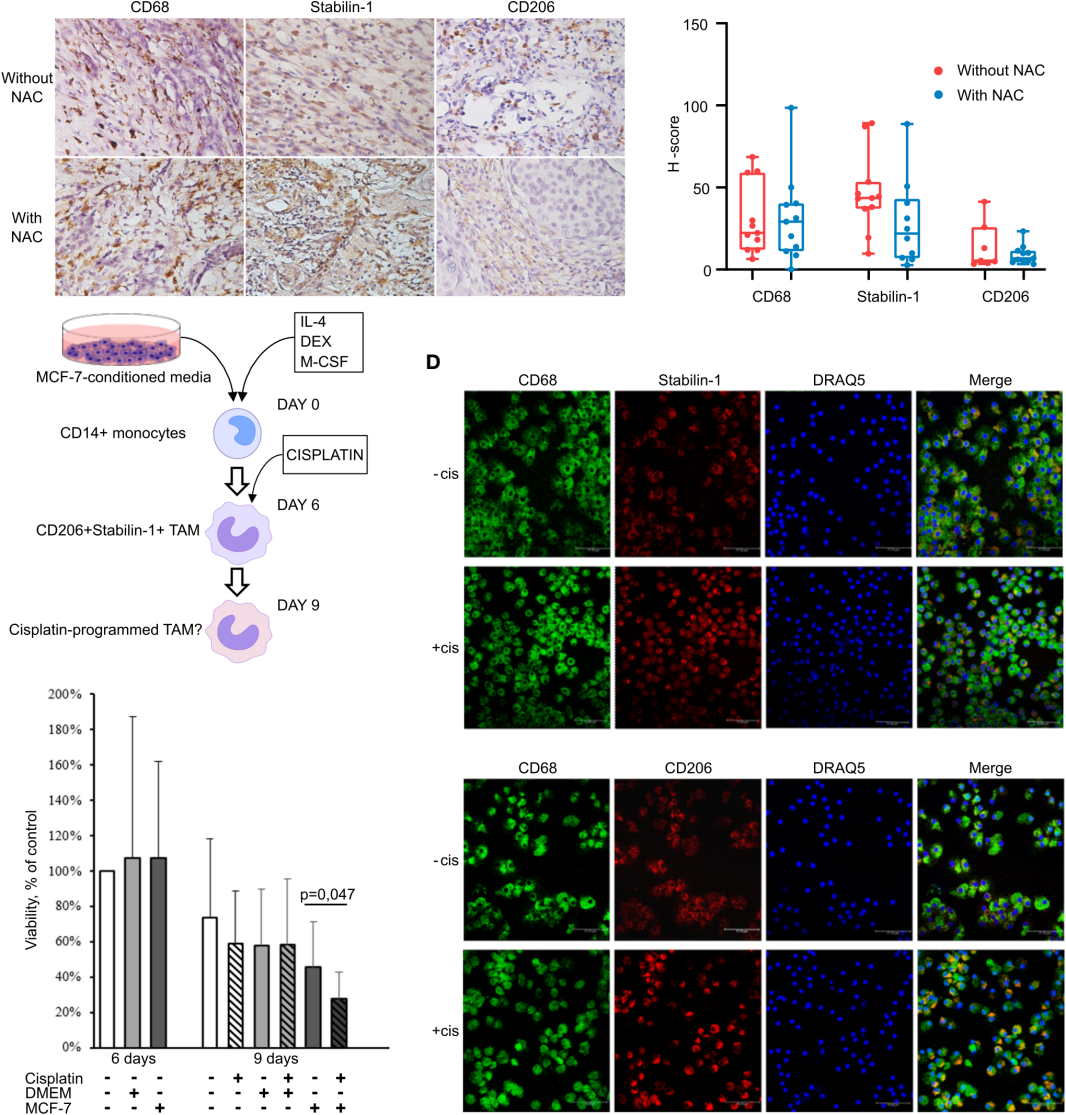
Cisplatin does not affect TAM viability and M2 phenotype. **(A)** IHC analysis of CD68, stabilin-1 and CD206 expression in human breast cancer tissue treated and not treated with cisplatin-based therapy. **(B)** Quantitative analysis of CD68, stabilin-1 and CD206 expression. Tumor tissue was obtained after surgery for all patients. **(C)** Schematic description of *ex vivo* TAM generation which were stimulated with MCF-7 supernatants and treated by cisplatin. **(D)** Immunofluorescent staining/confocal microscopy of CD206 and stabilin-1 expression in CD68+ *ex vivo* TAMs. Scale bars are equal to 11µM. **(E)** Viability assay for *ex vivo* TAMs treated with cisplatin. Viability was estimated on day 6 before cisplatin treatment and day 9 after cisplatin-treatment (n=6 independent experiments). Data information: In **(B)**, the level of protein expression is presented as Me (Q1;Q3). In **(E)**, OD means defined in the percentage of NS control are presented as mean ± SD. The Manna-Whitney test was applied to compare two independent groups.

We analyzed whether chemotherapeutic treatment can affect amount of TAMs in tumor tissue of patients with invasive breast carcinoma. Patients were divided into two groups according to the treatment: 1) patients who were not treated with neoadjuvant chemotherapy (NACT); and 2) patients underwent platinum-based NACT. The information about all patients is indicated in **Supplementary Table S1**. Chemotherapeutic treatment included CP (cisplatin plus cyclophosphamide) and CAP (cyclophosphamide, adriamycin and platinum) regimens based on cisplatin. The expression of CD68, stabilin-1, and CD206 was analyzed by quantitative IHC in these two groups. No statistically significant decrease in the amount of CD68+ and CD206+ macrophages was identified in the group of patients who underwent platinum-based NACT (**Figures 1A, B**). The same amount of stabilin-1+ macrophages was revealed in two groups (**Figure 1B**). We concluded that cisplatin-based treatment does not affect noticeably the amount of TAMs in breast cancer.

### Сisplatin induces TAM reprogramming

Since TAMs perfectly survive under cisplatin treatment in breast cancer patients, we raised the question whether cisplatin can affect TAMs programming and function. We developed TAM *ex vivo* model system where human monocyte-derived macrophages differentiate under tumor-specific conditions (**Figure 1C**). Conditioned supernatants from breast cancer (MCF-7) cells were used as a source of tumor-specific secreted factors to differentiate CD14+ monocytes into TAMs. IL-4, a known inducer of M2 polarization in the tumor microenvironment, was used to facilitate differentiation of monocytes towards tumor-associated phenotype (25). Differentiation of macrophages into the tumor-associated phenotype was confirmed by the expression of M2-like markers CD206 and stabilin-1 (7, 26) (**Figure 1D**). Confocal microscopy demonstrated that almost all CD68+ macrophages, differentiated in the presence of MCF-7 conditioned medium, expressed CD206 and stabilin-1. After 3 days of cisplatin treatment (at concertation of 20 µМ) 57% of TAMs were viable (**Figure 1E**).

**Figure 2:**
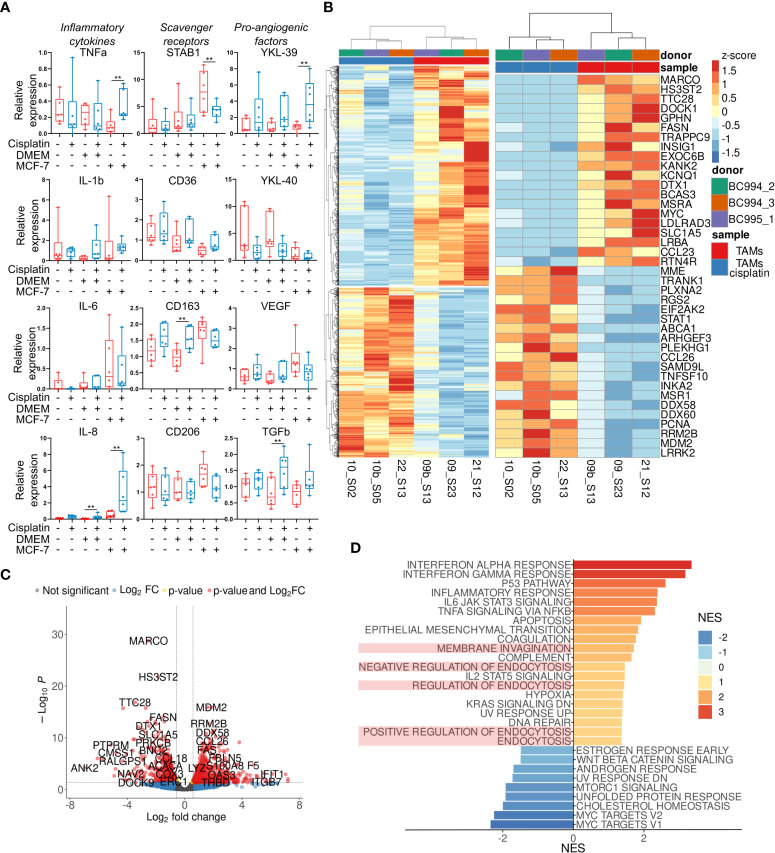
Transcriptional reprogramming of TAMs by cisplatin. **(A)** Effect of cisplatin on major inflammatory, scavenging and angiogenic factors analyzed by qPCR in *ex vivo* TAMs. Untreated – red, cisplatin-treated – blue (n=12 donors). **(B)** Results of RNA sequencing of *ex vivo* TAMs treated by cisplatin. Heatmap on the left depicts hierarchical clustering of upregulated and downregulated genes in *ex vivo* TAMs treated by cisplatin (p-value<0,05, log2 fold-change>0,58). On the right - heatmap with top 20 most DEGs induced by cisplatin. **(C)** Volcano plot shows significance and log2 fold-change value for DEGs in cisplatin-treated and untreated TAMs. **(D)** Bar plot with GSEA results demonstrates representative endocytosis pathways among other valuable pathways deregulated by cisplatin in *ex vivo* TAMs. The x-axis indicates NES (FDR<0,25, endocytosis pathways are highlighted in light-red). Data information: In **(A)**, the level of gene expression is presented as Me (Q1;Q3). The Manna-Whitney test was applied to compare two independent groups. **p ≤ 0.01.

The effect of cisplatin on the expression profile of *ex vivo* TAMs was quantified by RT-qPCR using selected biomarkers of TAM activity. We measured the gene expression levels of the spectrum of pro-inflammatory cytokines (TNFalpha, IL-1beta, IL-6, IL-8), scavenger receptors (STAB1, CD36, CD163, CD206), pro-angiogenic factors (TGFbeta, VEGFA), and chitinase-like proteins (YKL-39, YKL-40). The most pronounced effects of cisplatin on the gene expression profile of TAMs are illustrated by **Figure 2A**. The main stimulating effect of cisplatin was found for the expression of pro-inflammatory cytokines TNFa (fold change=3,62, p=0,024) and IL-8 (FC=5,92, p=0,024), and chitinase-like protein YKL-39, indicator for metastatic relapse (27) (FC=4,65, p=0,024). Recently in our laboratory, YKL-39 was found to be a novel strong pro-angiogenic factor (27). The transcription level of STAB1 statistically significantly decreased after cisplatin treatment (~1.7 fold, p<0.01). We could see difference in CD163 mRNA expression in TAMs stimulated with control culture media (DMEM), but this difference was not biologically significant. This data indicated that cisplatin does not eliminate TAMs, but changes their transcriptional program. This prompted us to perform full transcriptome analysis of TAMs in response to cisplatin by NGS.

### Transcriptome analysis of *ex vivo* TAMs induced by cisplatin

Our primary goal was to identify which tumor-supporting and tumor-inhibiting pathways are activated or suppressed by cisplatin in *ex vivo* TAMs. We performed whole transcriptome sequencing of 2 groups – *ex vivo* generated TAMs with and without cisplatin. About 22 million filtered reads were generated for each sample. Totally, 15050 filtered genes were analyzed. Differential expression analysis was performed by comparing TAMs with and without cisplatin. It allowed to identify 657 upregulated and 808 downregulated genes in TAMs under cisplatin (false discovery rate (FDR) <0.1, log2 fold-change>0.58) (**Figure 2B**). Representative top 35 most significant genes are demonstrated in **Figure 2B**. The volcano plot identified fold changes and significance for genes differentially expressed between TAMs with and without cisplatin treatment (**Figure 2C**).

We performed gene set enrichment analysis (GSEA) for up- and downregulated genes in cisplatin condition to evaluate pathway enrichment under cisplatin treatment. Pathways with normalized enrichment score (NES) > 1.30 and FDR<0.25 were identified. All genes were distributed in groups according to the biochemical and functional pathways using the following databases REACTOM, KEGG, HALLMARK, GO. The interferon alpha and gamma responses were the top upregulated pathways, along with p53 pathway, inflammatory response, IL-6 and TNFa signaling, epithelial-mesenchymal transition, endocytosis, hypoxia, KRAS signaling, DNA repair (**Figure 2D**). The downregulated genes showed significant enrichment for Myc targets, cholesterol homeostasis, mTORC1 signaling, WNTbeta-catenin signaling (**Figure 2D**).

We were interested in endocytic function of TAMs that was significantly affected by cisplatin. TAMs regulate composition and tumor-supporting activity of TME by SR-dependent clearance of extracellular mediators (28). We found that cisplatin affects gene expression of both stimulators and inhibitors of endocytosis (**Figure 2D**). We next focused on the endocytic function of stabilin-1. Stabilin-1 is a multifunctional scavenger receptor, which plays important roles in clearance of “unwanted” self-substances and remodeling of the TME (28). We previously showed that stabilin-1+ macrophages mediate efficient clearance of matricellular protein SPARC and contribute to tumor progression (29–31). We questioned if cisplatin could disturb the stabilin-1-mediated endocytosis in TAMs.

### Stabilin-1 mediates uptake of EGF in stably transfected CHO cells

**Figure 3:**
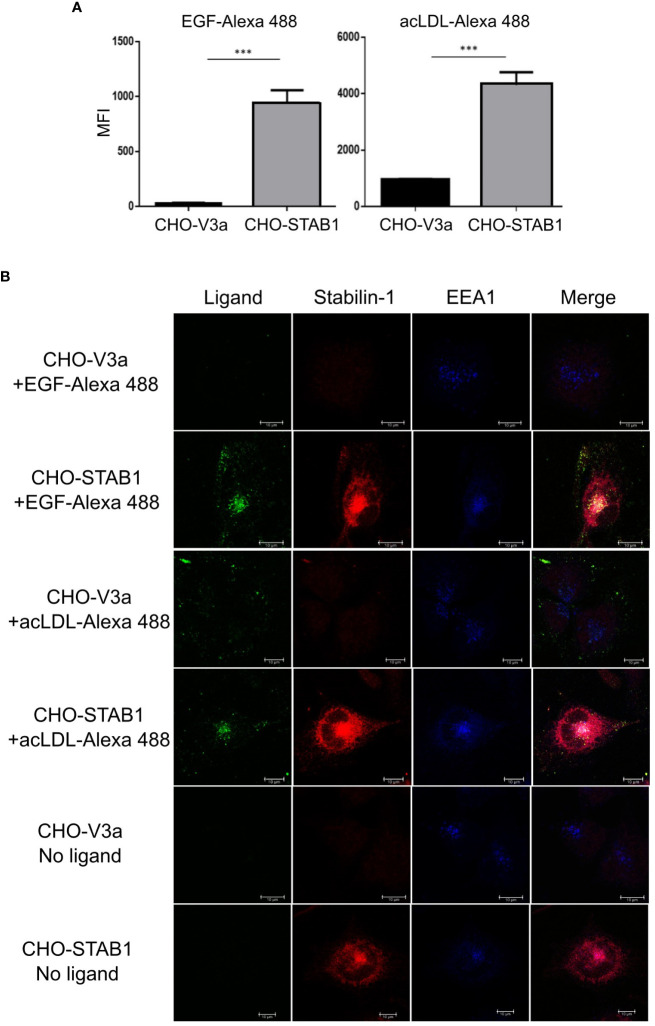
Stabilin-1 mediated endocytic uptake of EGF in CHO cells. **(A)** Flow cytometry analysis of acLDL and EGF uptake in CHO-Stab1 and CHO-vector control cells (CHO-V3a) (n=3). **(B)** Immunofluorescence/confocal microscopy of EGF-Alexa488 and acLDL-Alexa488 internalization in CHO-stabilin-1 and CHO-V3a. Yellow indicates co-localization of fluorescently labeled ligands and stabilin-1. White indicates co-localization of EGF-Alexa488 or acLDL-Alexa488, stabilin-1, and EEA-1. EGF-Alexa488 and acLDL-Alexa488 were internalized only in CHO-stabilin-1 cells. Scale bars: 10 µm. In (A), MFI index is presented as mean ± SD. *** - p<0,001. T-test was applied.

Stabilin-1, abundantly expressed on TAMs, has been identified by us as a scavenger receptor for a growth hormone family member placental lactogen and SPARC (19, 29). Here we examined whether stabilin-1 can mediate clearance of EGF, an essential growth factor that promotes breast cancer progression (32). We have used the model system of CHO cells ectopically expressing human stabilin-1, where we have previously identified scavenging function of stabilin-1 using acLDL, a ligand for stabilin-1 (18). Flow cytometry demonstrated efficient uptake of Alexa488-labeled EGF in CHO-stabilin-1 cells (approximately 10-fold) but not in the control CHO-vector cells. The endocytic activity of stabilin-1 was controlled by the uptake of acLDL-Alexa488 (**Figure 3A**).

Since flow cytometry signal can be produced not only by the receptor-mediated ligand internalization, but also by surface bound ligand retention, we examined the ability of stabilin-1 to target EGF to the endocytic pathway using confocal microscopy (**Figure 3B**). After 30 min of endocytosis, fluorescently-labeled EGF was detected in CHO-stabilin-1 cells, but not in CHO-vector control cells. EGF co-localized with stabilin-1 in EEA1-positive early/sorting endosomes, indicating that stabilin-1 mediates EGF trafficking to the endosomal pathway. The colocalization of stabilin-1 and EGF was similar to acLDL, classical endocytic ligand of stabilin-1 (**Figure 3B**). Thus, for the first time we identified that stabilin-1 acts as a scavenger receptor for internalization and endocytic trafficking of EGF, an essential regulator of tumor growth.

### Cisplatin impairs endocytosis of EGF and acLDL in *ex vivo* TAMs

**Figure 4:**
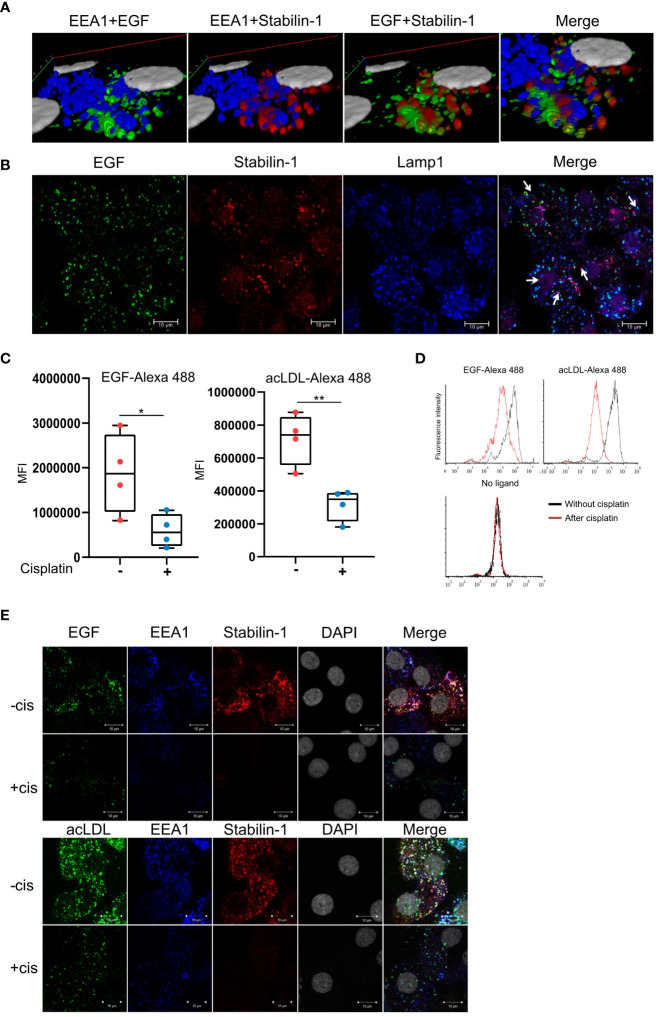
Cisplatin inhibits EGF clearance by human TAMs. **(A)** 3D reconstruction of Z-stack confocal microcopy images for EGF and stabilin-1 co-localization in EEA1+ endosomes in TAMs **(B)** Confocal microscopy analysis of EGF transfer to Lamp1+ lysosomes after 30 min of endocytosis in TAMs. **(C)** Flow cytometry analysis of endocytosis of EGF and acLDL in *ex vivo* TAMs treated with cisplatin (n=4 donors). **(D)** Representative flow cytometry diagrams of ligand uptake. The fluorescent intensity is significantly lower in TAMs, treated with cisplatin. **(E)** Immunofluorescent/confocal analysis of stabilin-1 mediated internalization and trafficking of EGF and acLDL in TAMs without and with cisplatin treatment. White indicates co-localization of fluorescently labeled ligand, stabilin-1, and EEA-1 (n=4 donors). Scale bars: 10µM. Data information: In **(C)**, MFI index is presented as Me (Q1;Q3), * - p<0,05, ** - p<0,01. The Manna-Whitney test was applied to compare two independent groups.

We analyzed the effect of cisplatin on scavenging function in *ex vivo* TAMs. First, we demonstrated that stabilin-1 is co-localized with EGF-AlexaFluor488 in EEA1+ endosomes in TAMs that was shown by Z stack image acquisition and 3D visualization (**Figure 4A**). We also demonstrated that part of internalized EGF-AlexaFluor488 is already transferred to Lamp-1 positive lysosomes after 30 min of endocytosis by TAMs (**Figure 4B**). In order to assess the effect of cisplatin on scavenging function of TAMs, the acLDL was used as a general ligand of scavenging receptors, and EGF was used as a tumor-specific ligand. After 30 min exposure, the uptake of acLDL-AlexaFluor488 or EGF-AlexaFluor488 by TAMs was quantified by flow cytometry (**Figures 4C, D**). Cisplatin statistically significantly suppressed uptake of EGF and classical stabilin-1 ligand acLDL in TAMs (FC=2,25, p<0,01, and FC=5,4, p=0,023, correspondingly) (**Figure 4C**). At the same time, we found that the expression of stabilin-1 analyzed by real-time qPCR statistically significant decreased after cisplatin treatment in *ex vivo* TAMs (**Figure 2A**).

We next analyzed stabilin-1-mediated internalization of acLDL and EGF in *ex vivo* TAMs after cisplatin treatment. Trafficking of fluorescently labeled ligands to the EEA1-positive early/sorting endosomes was visualized by immunofluorescence/confocal microscopy. In the absence of cisplatin, acLDL and EGF were efficiently internalized by *ex vivo* generated TAMs. Both acLDL and EGF were detected in EEA1+ early endosomes, where they also co-localized with stabilin-1, confirming its involvement in EGF endocytosis in macrophages. Treatment with cisplatin resulted in the decrease of the internalization and delivery of both acLDL and EGF to the EEA1+ early endosomes (**Figure 4E**). Our data indicated that cisplatin interferes with the receptor-mediated ligand delivery to the endosomal system in TAMs. Confocal microscopy analysis of the ligand internalization demonstrated that amount of stablin-1 protein is in excess to the ligand that has to be internalized, and is not limiting for the endocytic uptake (**Figure 4E**). Therefore, we have further searched for the molecular mechanism that restricts endocytic activity of TAMs after cisplatin treatment.

### Сisplatin causes the defects in endocytic machinery by reducing membrane biogenesis and vesicular transport

**Figure 5:**
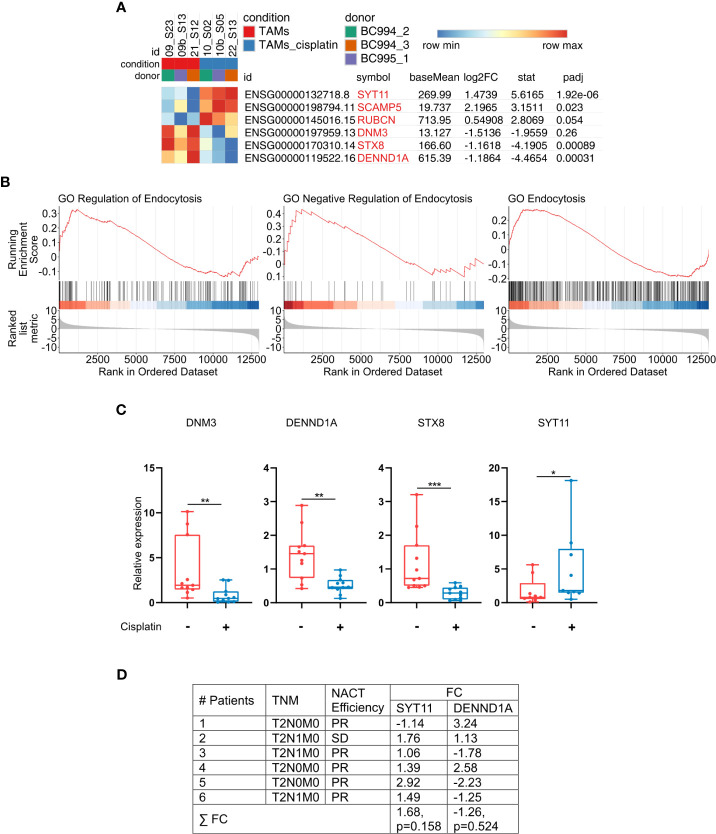
Cisplatin induces imbalance in the endocytic machinery of TAMs. **(A)** Gene set from endocytosis pathways (RT-PCR validated genes are highlighted in light-red). Results are obtained with RNAseq. **(B)** GSEA enrichment plots illustrating GO endocytosis pathways. The y-axis indicates enrichment score, the x-axis represents gene ranks (FDR<0,1, FDR<0,25, respectively). **(C)** Cisplatin suppresses expression of genes, that positively regulate endocytosis and vesicular transport, and stimulates expression of negative regulator of endocytosis (*SYT11*). *DNM3, DENND1A*, *STX8* and *SYT11* were analyzed by qPCR in *ex vivo* TAMs (n=12). **(D)** Differential *SYT11* and *DENND1A* expression in patients` breast cancer samples identified by microarray analysis. Breast cancer tissues before and after cisplatin-based NACT were compared. PR – partial regression, SD – stable disease, FC – Fold Change. FC defines the differences in the pre- and post-treatment levels of gene expression. Data information: In **(C)**, the level of gene expression is presented as Me (Q1;Q3), * - p<0,05, **p ≤ 0.01, *** - p<0,001. Untreated – red, cisplatin-treated – blue. Manna-Whitney test was applied to compare two independent groups.

**Figure 6:**
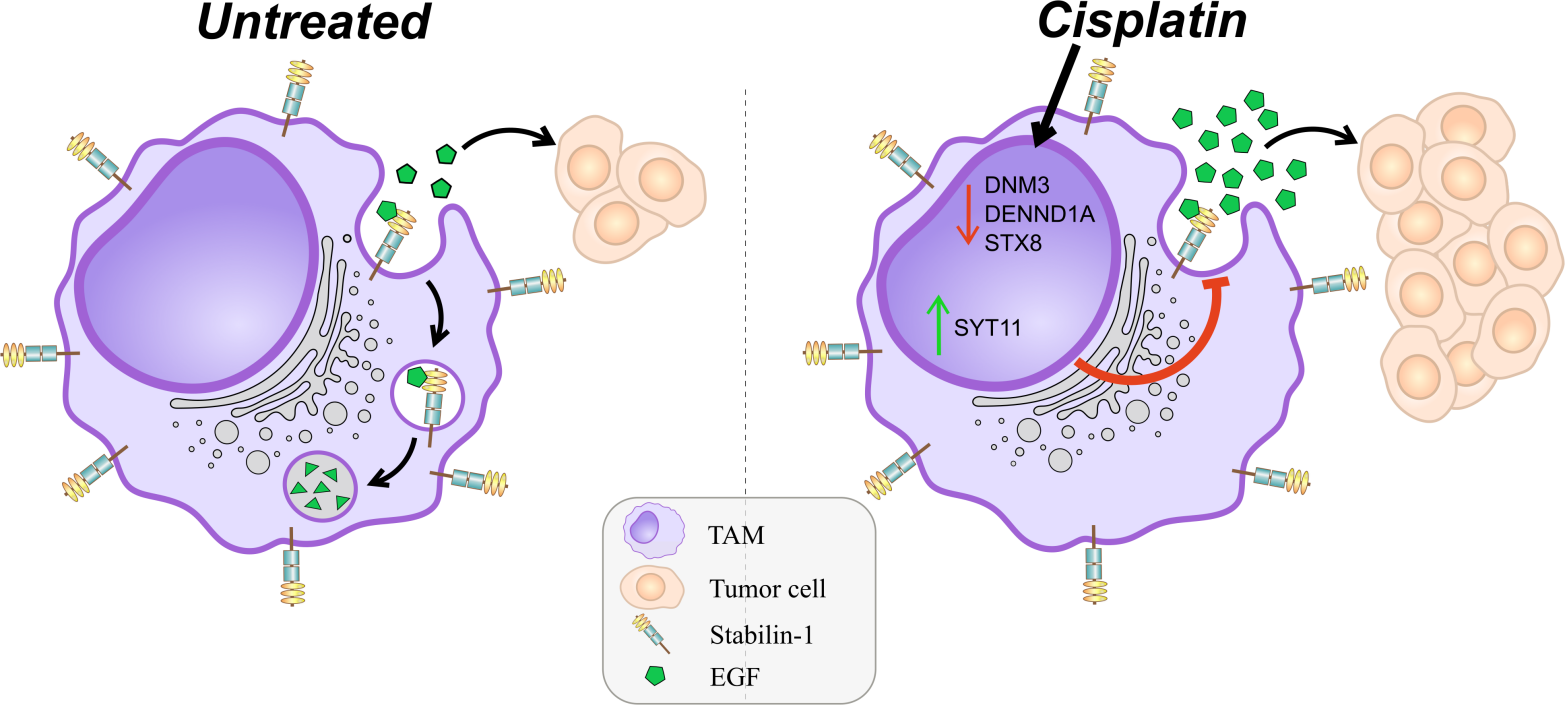
Schematic presentation of detrimental effect of cisplatin on TAMs. M2 scavenger receptor stabilin-1 remains to be expressed after cisplatin treatment. Stabilin-1 is able to clear EGF from breast cancer TME specifically. Cisplatin suppresses endocytic vesicular machinery by dual effect. The first effect is suppression of the expression of positive regulators of endocytic membrane biogenesis (STX8, DENND1A, DNM3). The second effect is stimulation of the expression of negative regulator of endocytosis (SYT11). Combination of such cisplatin effects results in significantly impaired receptor-mediated endocytic clearance of soluble simulators of tumor growth.

Since we showed that cisplatin decreases the uptake and endocytic trafficking of both classical ligand LDL and tumor-specific ligand EGF but does not significantly affect the SR expression, next we asked which mechanisms are involved in this process. Using NGS data, we identified that cisplatin treatment leads to the dysregulation of genes, involved in the following process: vesicle mediated transport, endosomal sorting complex required for transport, clathrin-derived vesicle budding, trans-Golgi network vesicle budding and other related processes. Validation of sequencing data by real-time qPCR was performed for selected genes implicated in the endocytic uptake: DNM3, STX8 and DENND1A, which are downregulated by cisplatin and enhance endocytosis and vesicular transport, and SYT11, SCAMP5 and RUBCN, which are upregulated by cisplatin and have negative effect on endocytosis (**Figures 5A, B**). Statistically significant downregulation in the gene expression was found for positive regulators of endocytosis: DNM3, STX8, DENND1A in *ex vivo* TAMs of independent samples (**Figure 5C**). The most pronounced decrease in response to cisplatin treatment was detected for DNM3, GTPase needed for the efficient ligand internalization (FC=-4,34, p=0,005) (**Table 1** and **Figure 5C**). In contrast, cisplatin treatment resulted in the elevated expression of negative regulator of endocytosis: SYT11 (**Table 1** and **Figure 5C**). We did not find statistically significant differences for RUBCN and SCAMP5 expression. The expression of SYT11, which inhibits endocytosis, was almost 3 times higher in cisplatin-treated TAMs (FC=2,97, p=0,037). The detailed mechanism of cisplatin action on TAMs is illustrated by **Figure 6**.

**Table 1:** The functions and parameters of differential expression of genes involved in endocytosis affected by cisplatin treatment.

Gene name	FC, padj-value (NGS data)	FC, p-value (real-time PCR data)	Biological function
DNM3 (dynamin 3)	FC=-2,85; padj=0,263	FC=-4,344 **p=0,005**	Dynamin is a multi-domain GTPase. Dynamin interacts with proteins involved in coordination of endocytosis with motor molecules, and vesicle transport. It is required for efficient ligand internalization supporting actin-associated proteins (33, 34).
STX8 (syntaxin 8)	FC=-2,23; **padj=0,000892**	FC=-4,085 **p=0,0003**	STX8 is localized to the trans-Golgi network and to the early and late endosomes. It is involved in the internalization from the plasma membrane and the protein trafficking from early to late endosomes *via* vesicle fusion 59 (35).
DENND1A (connecden)	FC=-2,27; **padj=0,000305**	FC=-2,732 **p=0,002**	DENND1A functions as guanine nucleotide exchange factors (GEFs) regulating clathrin-mediated endocytosis through the early endosomal small GTPase RAB35 and binding to clathrin and clathrin adaptor protein-2 (36).
SYT11 (synaptotagmin IX)	FC=2,77; **padj=1.92e-06**	FC=2,977 **p = 0,037**	Ca2+ sensor for SNARE‐dependent vesicle fusion; inhibits clathrin‐mediated and bulk endocytosis (37, 38).
SCAMP5	FC=4,59; **padj=0,0231**	FC=2,330 p = 0,565	Regulates endocytosis by recruiting EH-domain proteins to the N-terminal NPF repeats (39).
RUBCN	FC=1,46; padj=0,0538	FC=2,022 p = 0,141	A Beclin 1-binding protein, negatively regulates both the autophagic and endocytic pathways by interacting with Rab7 *via* RH domain (40).

Statistically significant p-values are marked in bold.

### SYT11 expression is enhanced by NACT in breast cancer patients

To validate the data obtained in *ex vivo* system we have performed whole-transcriptome Affimetrix microarray in clinical samples obtained from breast cancer patients treated with cisplatin-based NACT. For all patients we compared tumor biopsy specimens taken before NACT and remaining tumor in post-surgery specimens after NACT. We found that expression of SYT11 was elevated in this patients’ group after NACT (**Figure 5D**). The effect of NACT differed between patients, with the highest increase in patient 5 (almost 3 times). This data provides the validation for the cisplatin mode of transcriptional program identified in our model system.

## Discussion

Despite significant progress in the development of novel therapeutic methods, successful treatment of cancer still requires personalized approaches (14). Therapeutic sensitivity of tumors depends on the intrinsic mechanisms inside tumor cell and their cross-talk with different components of tumor microenvironment, in particular TAMs. Our study answers urgent question of the direct response of TAMs to widely used chemotherapeutic agent cisplatin.

Cisplatin is a platinum-based DNA-intercalating agent that forms DNA crosslinks (16). Cisplatin therapy has been suggested for triple-negative BC harboring a BRCA mutation (41). The complete pathological responses in BC patients was achieved in numerous clinical studies (41, 42). However, despite an initial response, cisplatin treatment results in the development of chemoresistance in patients (43). TAM targeting was proposed as a possible approach to improve the efficacy of platinum-based therapy (44). Recently, Salvagno and colleagues showed that CSF-1R blockade stimulates an intratumoral type I IFN response and enhances cisplatin anti-tumor effect in transgenic mouse model of BC (44).

We examined clinical samples of patients with breast cancer to analyze whether NACT results in any significant decrease of the amount of TAMs. By quantitative immunohistochemistry we found that cisplatin-based NACT has not resulted in the biologically significant decrease of total amount of CD68+ TAMs and amount of CD206+ and stabilin-1+ M2-like TAMs in the remaining tumor tissue compared to the non-treated tumor. Similarly, in the *ex vivo* system of human TAMs, cisplatin treatment did not significantly affect viability of macrophages. Our initial real-time qPCR analysis of selected biomarkers of TAM activation revealed that cisplatin can both stimulate and suppress expression of secreted and transmembrane factors, showing that TAMs change their program toward a mixed inflammatory program, and can alter balance in pro-angiogenic and scavenging programs. Isolated reports previously noted that there is an effect of cisplatin on the macrophage polarization. Liu W et al. have detected increasing level of IL-1b in classically activated THP1 and PBMC-derived human macrophages (45). However, the authors believed that the effects of cisplatin on macrophage programming is minor.

In order to identify the full transcriptional program induced by cisplatin in macrophages, we developed an *ex vivo* model of human TAMs that resemble phenotypic and functional characteristic of TAMs in breast cancer. We performed whole-transcriptome sequencing (NGS) and demonstrated profound changes in the transcriptional program in macrophages treated by cisplatin.

We decided to focus on the endocytic function of macrophages, which deserve specific attention in context of cancer.

To perform highly efficient and selective scavenging functions, macrophages are equipped with the endocytic receptors - heterogeneous transmembrane proteins with complex extracellular domains recognizing broad range of non-self and unwanted-self ligands including apoptotic bodies and modified lipoproteins (9, 46). Expression of endocytic receptors is also indicative for the functional polarization of macrophages, and in human tumors stabilin-1, CD163, CD206, CD204, MARCO are most frequently used to distinguish diverse functional TAM polarization (7). The endocytosis of cancer-associated ligand by a scavenger receptor on macrophages was previously shown by us. We found that alternatively activated macrophages utilize stabilin-1 for the efficient clearance of soluble component of tumor extracellular matrix SPARC (29).

In this study for the first time we show that stabilin-1 is a specific scavenger receptor for EGF, a growth factor critical for progression of breast cancer (47, 48). The hypothesis about stabilin-1 involvement in EGF internalization was based on our previous findings that stabilin-1 performs endocytic clearance of growth hormone family member placental lactogen and growth/differentiation factor 15 (GDF-15), while, to our knowledge no other SRs were reported (19, 46, 49, 50). In breast cancer stabilin-1 is predominantly expressed on TAMs, and not on other cells types (like lymphatic, angiogenic or sinusoidal endothelial cells) (31, 51–54).

EGF is highly efficiently and specifically endocytosed by stabilin-1 and the efficiency of the internalization was similar to that previously found for growth hormone family member placental lactogen (PL) (19). Thereafter, we have demonstrated that the uptake of general ligand of scavenging receptors acLDL and tumor-specific ligand EGF in TAMs of breast cancer was significantly decreased in the presence of cisplatin. EGF is expressed by many types of tumors. The signaling pathway driven by EGF/EGFR is an important regulator of tumor growth, invasion and metastasis in epithelial malignancies (47). EGF was shown to induce epithelial-mesenchymal transition (EMT) in EGFR-expressing human breast carcinoma cells (47). Since EGF is a significant positive regulator of tumor growth, the disruption of receptor-depending clearance of such tumor-supportive factor by chemotherapeutic drug may lead to the macrophage-mediated homeostatic imbalance in TME. One of the possible mechanisms of drug resistance is a clonal expansion of chemoresistant clones of tumor due to inability of TAMs to uptake tumor growth factors (55). Li et al. showed that EGF stimulation of androgen-independent human prostate cancer cells DU145 and PC-3 exhibited stronger resistance to cisplatin (56).

The essential finding we made was that cisplatin has bidirectional mechanism to suppress endocytosis in TAMs. NGS confirmed by RT-PCR demonstrated that cisplatin suppresses positive regulators of endocytosis and membrane transport: DNM3, STX8, DENND1A. At the same time, cisplatin upregulates the expression of SYT11, that inhibits endocytosis (**Table 1**). Cisplatin-mediated suppression of positive regulators of endocytosis can inhibit vesicle formation, impair TAM clearance function in TME and enhance tumor resistance to chemotherapy. Similarly, Perrotta and co-authors showed that TAMs are key factors limiting tumor cell sensitivity to cisplatin. Cisplatin resistance was mediated by the inhibiting STX4, which plays role in the translocation of tumor suppressive protein A-SMase to the plasma membrane and its activation (55).

The strongest suppressing effect of cisplatin was found for SYT11, a non-Ca2+-binding synaptotagmin, that inhibits clathrin‐mediated endocytosis and bulk endocytosis in dorsal root ganglion neurons and in microglia (57, 58). SYT11 also acts as a negative regulator for cytokine secretion by affecting SNARE complex in macrophages (59). We were able to confirm the data obtained in the model TAMs it tumor samples of patients with breast cancer, and found enhancing effects of cisplatin on SYT11 expression.

Our data demonstrated that cisplatin affects important elements of endocytic machinery in TAMs that correlates with impaired endocytic clearance of tumor supporting factor EGF from TME. Identified mechanism may be used as a target to enhance efficiency of chemotherapy. In breast cancer, tumor heterogeneity is an essential factor that defines tumor development and tumor response to therapy (2). In future, pivotal progress can be achieved if the interaction of TAMs with therapeutic agents is examined in the context of intratumoral heterogeneity, and key localizations where TAMs define therapy resistance are identified. Understanding the fundamentally important role of TAMs in the tumor response to cytostatic therapy will open the prospect of developing new therapeutic approaches for the treatment of malignant diseases based on the balanced synergistic action of cytostatic agents and innovative immunomodulatory approaches.

## Data availability statement

The data generated in this study are available within the article and its supplementary data files. Results of RNAseq analysis are presented in NCBI data portal (GSE224681): https://www.ncbi.nlm.nih.gov/geo/query/acc.cgi?acc=GSE224681.

## Ethics statement

The study was carried out according to Declaration of Helsinki (from 1964, revised in 1975 and 1983) and was approved by the local committee of Medical Ethics of Tomsk Cancer Research Institute. The patients/participants provided their written informed consent to participate in this study.

## Author contributions

Conceptualization: IL and JK; data curation: IL and JK; formal analysis: IL, EK, MP, NL, MT, MI, NC, TL and NB; validation: IL and EK; investigation: IL, EK, PI, MP, DM, VR, MR, SV, ED, PK, MT, MI and QL; visualization: EK and PI; methodology: IL, JK and DP; writing–original draft: IL, EK, MP, PI, and NL; resources: AK, PI and AS; supervision, funding acquisition, and directed the project: JK. All authors contributed to the article and approved the submitted version.
