# Supplementary material for: Tumor-associated macrophages respond to chemotherapy by detrimental transcriptional reprogramming and suppressing stabilin-1 mediated clearance of EGF
Source: Front Immunol. 2023 Mar 7;14:1000497. doi: 10.3389/fimmu.2023.1000497 (PMC10028613; doi:10.3389/fimmu.2023.1000497)
Supplement: Supplementary file 1 [file Table_1.docx]

Supplementary information

Table S1. Clinical parameters of patients involved in IHC study

| Number of patient | NAC | NAC response | Stage | TNM classification | Molecular subtype |
| --- | --- | --- | --- | --- | --- |
| 1 | CP | PR | 2 | T_2_N_0_M_0_ | luminal B |
| 2 | CP | PR | 2 | T_2_N_1_M_0_ | luminal B |
| 3 | CAP | PR | 2 | T_2_N_1_M_0_ | luminal B |
| 4 | CP | PR | 2 | T_2_N_0_M_0_ | BRCA1-associated TN |
| 5 | CP | S | 2 | T_2_N_1_M_0_ | luminal B |
| 6 | CP | PR | 2 | T_2_N_0_M_0_ | BRCA1+ TN |
| 7 | CP | PR | 2 | T_2_N_0_M_0_ | BRCA1+ luminal B |
| 8 | CP | PR | 2 | T_3_N_1_M_0_ | BRCA1+ luminal B |
| 9 | CAP | PR | 1 | T_1_N_0_M_0_ | luminal B |
| 10 | CAP+CP | S | 3 | T_3_N_3_M_0_ | BRCA1+ TN |
| 11 | CAP | PR | 2 | T_2_N_0_M_0_ | BRCA1+ TN |
| 12 | no | - | 1 | T_1_N_0_M_0_ | TN |
| 13 | no | **-** | 2 | T_1_N_0_M_0_ | luminal A |
| 14 | no | **-** | 3 | T_4_N_1_M_0_ | luminal B |
| 15 | no | **-** | 1 | T_1_N_0_M_0_ | TN |
| 16 | no | **-** | 1 | T_1_N_0_M_0_ | luminal B |
| 17 | no | **-** | 2 | T_2_N_0_M_0_ | luminal B |
| 18 | no | **-** | 3 | T_4_N_1_M_0_ | luminal B |
| 19 | no | **-** | 2 | T_2_N_1_M_0_ | luminal A |
| 20 | no | **-** | 1 | T_1_N_0_M_0_ | luminal B |
| 21 | no | **-** | 2 | T_2_N_1_M_0_ | TN |
| 22 | no | **-** | 1 | T_1_N_0_M_0_ | luminal B |
| 23 | no | **-** | 2 | T_2_N_0_M_0_ | luminal A |
| 24 | no | **-** | 1 | T_1_N_0_M_0_ | TN |
| 25 | no | **-** | 2 | T_2_N_0_M_0_ | luminal A |
| 26 | no | **-** | 2 | T_2_N_1_M_0_ | luminal B |
| 27 | no | **-** | 2 | T_2_N_1_M_0_ | luminal B |

Notes: CAP – cyclophosphamide, adriamycin and platinum; CP – cisplatin plus cyclophosphamide) NAC – neoadjuvant chemotherapy; PR – partial response; S – stabilization; TN – triple-negative.

**Table S2. Sequences of primers with original design used for quantitative real-time PCR analysis**

| **Gene** | **Amplicon** | **Sequence** |
| --- | --- | --- |
| *GAPDH*  NM_002046.3 | 124 bp | F 5'-gccagccgagccacatc-3' |
|  |  | R 5'-ggcaacaatatccactttaccaga-3' |
| DNM3  NM_015569.5 | 89 bp | F 5’-gagatagtaaagatggagttcaatga-3’ |
|  |  | R 5’-aaccctgtcctgataccatgta-3’ |
| STX8  NM_004853.3 | 93 bp | F 5’-gtcaacacatcagataacacagct-3’ |
|  |  | R 5’-aaggatgccagaagtagtctctc-3’ |
| DENND1A  NM_001352966.1 | 80 bp | F 5’-catctcagcgtgcattcttat-3’ |
|  |  | R 5’-tctgtcagatttctattctcaggtat-3’ |
| SYT11  NM_152280.5 | 118 bp | F 5'-acaactgaccagggacatca-3' |
|  |  | F 5'-tttgaggaccaccactgtca-3' |
| SCAMP5  NM_138967.4 | 119 bp | F 5'-cagttcaggacaaagaggtgtg-3' |
|  |  | F 5'-tggtagaaacatggcttcagc-3' |
| RUBCN  NM_014687.4 | 110 bp | F 5'-cctggaagcagtggaacaga-3' |
|  |  | F 5'-tcaggctctggctctttgtc-3' |
| STAB1  NM_015136 | 98 bp | F5’-tccgcctcctggaatataag-3’ |
|  |  | R5’-cagctcatcctgcgacag-3’ |

Notes: all probes – FAM →BHQ1; NM – number of RNA sequence in NCBI Nucleotide Database (<http://www.ncbi.nlm.nih.gov/nuccore>); bp – base pair; F – forward primer; R – reverse primer. All primers have the original design.
